# Molecular evolution and population genetics of a Gram-negative binding protein gene in the malaria vector *Anopheles gambiae* (*sensu lato*)

**DOI:** 10.1186/s13071-016-1800-2

**Published:** 2016-09-23

**Authors:** Patrícia Salgueiro, Ana Sofia Lopes, Cristina Mendes, Jacques Derek Charlwood, Ana Paula Arez, João Pinto, Henrique Silveira

**Affiliations:** 1 Global Health and Tropical Medicine Centre (GHTM), Unidade de Parasitologia Médica, Instituto de Higiene e Medicina Tropical (IHMT), Universidade Nova de Lisboa, Lisboa, Portugal; 2 London School of Hygiene and Tropical Medicine, London, UK

**Keywords:** *Anopheles gambiae*, *Anopheles coluzzii*, Gram-negative binding protein gene, Glucan binding protein gene, Innate immune system

## Abstract

**Background:**

Clarifying the role of the innate immune system of the malaria vector *Anopheles gambiae* is a potential way to block the development of the *Plasmodium* parasites. Pathogen recognition is the first step of innate immune response, where pattern recognition proteins like GNBPs play a central role.

**Results:**

We analysed 70 sequences of the protein coding gene *GNBPB2* from two species, *Anopheles gambiae* (*s.s.*) and *An. coluzzii*, collected in six African countries. We detected 135 segregating sites defining 63 distinct haplotypes and 30 proteins. Mean nucleotide diversity (π) was 0.014 for both species. We found no significant genetic differentiation between species, but a significant positive correlation between genetic differentiation and geographical distance among populations.

**Conclusions:**

Species status seems to contribute less for the molecular differentiation in *GNBPB2* than geographical region in the African continent (West and East). Purifying selection was found to be the most common form of selection, as in many other immunity-related genes. Diversifying selection may be also operating in the *GNBPB2* gene.

**Electronic supplementary material:**

The online version of this article (doi:10.1186/s13071-016-1800-2) contains supplementary material, which is available to authorized users.

## Background

In order to complete their life-cycle, the malaria parasites *Plasmodium sp*. have to go through important stages within their mosquito vectors *Anopheles sp.*, before being transmitted to human hosts. Malaria control strategies based on obstructing the parasite life-cycle within the mosquito are dependent on an understanding of the mosquito anti-pathogen defence system [1, 2]. This has been facilitated by the availability of the *Anopheles gambiae* genome sequence [3, 4].

The mosquito innate immune system constitutes a major barrier to infection [5, 6]. The first step of the innate immune response is pathogen recognition, which is activated by pattern recognition receptors (PRRs) that bind to pathogen-associated molecular patterns [7]. One important group of PRRs are the Gram-negative bacteria-binding proteins or glucan-binding proteins (GNBPs). These were initially identified in *An. gambiae* due to the similarities with GNBPs from other insects and because they are transcriptionally upregulated following infection with bacteria and *Plasmodium* parasites [8]. Six members of this gene family are expressed in *An. gambiae* and function as PRRs by binding ß-1,3-glucan and lipopolysaccharide on the surface of pathogens.

GNBPs are divided into two distinct sequence groups: subfamily A, that includes all known fruit fly and moth as well as two mosquito sequences (*GNBPA* 1 and 2); and subfamily B that is mosquito-specific (*GNBPB* 1, 2, 3 and 4) and probably a result from gene duplication [7].

Prior work reported that GNBPs are transcribed in multiple tissues (hemocytes, midgut, and salivary glands) and while they are all upregulated following an immune challenge, they vary in their antimicrobial specificities [7–14]. Specifically, GNBPs have been shown to regulate immune gene expression through the Toll or the IMD (Immune Deficiency) pathways. Certain members are able to mediate *Plasmodium* oocyst intensities in *An. gambiae* [13] and one GNBP homologue in *An. gambiae* was highly expressed in the fat body and salivary glands [8, 15]. On the other hand, in cultured *Anopheles* cells infected with *Wolbachia* strains, *GNBPB1* gene was downregulated by *Wolbachia* infection [16]. As for *GNBPB2*, it has been shown to be induced by challenges with *Salmonella typhimurium* [13] and *Beauveria bassiana* [11]. It was also upregulated upon challenge with *Escherichia coli* [13].

In sub-Saharan Africa, most malaria transmission is sustained by members of the *Anopheles gambiae* complex. Within the nominal species two molecular forms (denoted M and S) were previously described (see [17] and references therein). Recently, these molecular forms were reclassified as distinct species, and the M-form was named *Anopheles coluzzii*, while the S form retained the nominotypical name *An. gambiae* [17].

Early genome-wide genotyping studies have shown that most of the genetic divergence between *An. gambiae* (*s.s*.) and *An. coluzzii* is concentrated in three relatively small centromeric regions in X, 2L and 3L [18–20]. Differentiation was also detected in immunity genes between the two species [21–23] with the most remarkable case being the near fixation in *An. coluzzii* of an allelic variant of the thioester-containing protein 1 (TEP1) [23]. TEP1 is an important component in the innate immune response of *An. gambiae* to *Plasmodium* infection, which targets malaria parasites for destruction during their initial invasion of the body cavity. Several studies have addressed the molecular evolution and genetic diversity of the anti-malaria immune genes of *An. gambiae* [21, 22, 24–33]. However, only a few have focused on *GNBP* genes [21, 24, 26, 30, 34].

In order to untangle the modes of selection operating in the gene GNBPB2 and better understand its evolution in malaria vectors, patterns of genetic diversity and population differentiation were examined in samples of *An. gambiae* (*s.s*.) and *An. coluzzii* from six sub-Saharan African countries.

## Methods

### Mosquito sampling

Mosquito samples analysed in this study were collected mainly indoors by various methods of adult sampling during the rainy season in seven localities from six sub-Saharan African countries, within the framework of epidemiological surveys. Details on these collections can be found in Additional file 1: Table S1. After collection, individual specimens were kept in silica gel filled tubes.

### DNA extraction, PCR amplification and sequencing

Genomic DNA was extracted from each specimen as described in Collins et al. [35]. Species identification of the members of the *An. gambiae* complex was carried out by PCR-RFLP as described in Favia et al. [36].

The primers used to amplify the *GNBPB2* gene were designed based on the complete *An. gambiae* genome at Ensembl (sequence annotated AGAP002798). These primers are described in Table 1 and available at NCBI Probe database (Pr032290638).

**Table 1:** Primer sequences used to amplify the GNBPB2 gene (NCBI Probe database accession number: Pr032290638)

PCR reaction	Primer	Sequence 5′-3′	Product size (bp)
1	GNBPB2-out-F	CACTCCAGCGAACATTTGTG	1902
	GNBPB2-out-R	CTTCAGTGTGTGGCGGTTTA	
2	GNBPB2-in-3-F	CCCTAAATAAAGCGGCACAC	851
	GNBPB2-in-3-R	GCACTCTTGATGGGGTTGAT	
3	GNBPB2-in-5-F	GTTCTGGGGATGTGAGCGTA	963
	GNBPB2-in-5-R	CAGGGATCTTTTGCGTGATT	
4	GNBPB2-centre-F	ACRGGAGAGCTGATCTTTGA	571
	GNBPB2-centre -R	GCCWCGRTAGTCCATATTGC	

Nested PCR assays were performed in 50 μl reaction volumes with final reagent concentrations of 1× reaction buffer, 3 μM of MgCl_2_, 4 μM dNTPs, 0.5 μM of each primer (except for the *centre primers* with 0.1 μM), and 0.05 U/μl of Taq DNA polymerase. PCR cycling conditions consisted in 2 min of initial denaturation at 95 °C, followed by 35 cycles of 1 min at 95 °C, 30 s at 51 °C, 1 min at 72 °C and a final extension step of 5 min at 72 °C. For the *primers out,* the intermediate step of 72 °C lasted for 2 min. For the *primers centre*, the annealing temperature was 55 °C. PCR products were purified with SureClean kit (BIOLINE, London, UK) and commercially sequenced by Macrogen, Korea.

### Data analysis

Sequences were edited and aligned with BioEdit Sequence Alignment Editor version 7.0.5.2 [37]. In DnaSP version 5.10.01 [38] the different coding and non-coding regions were defined and the translated sequences were obtained.

Summary statistics, including the number of segregating sites (S), number of haplotypes (Hap), Haplotype diversity (Hd), nucleotide diversity (π), and the standard neutrality tests: Tajima’s D [39], Fu and Li’s D* and F*[40] and Fu’s Fs [41] were calculated using DnaSP. This program was also used to compute π between species and the π(a)/π(s) ratios along the gene *GNBP2* using the sliding window option (window length = 50 bp; sliding interval = 10 bp).

Additionally, we performed the dN/dS test for detecting selection implemented in the HYPHY program [42]. This test was also executed in a new alignment with the original sequences of the exon 2 obtained in the present study, to which 31 sequences of the same exon (599 bp) of *An. gambiae* from Cameroon available on GenBank (accession numbers AM774987–AM774989, AM774998–AM775011 [21, 34], AM900863–AM900876 [34]) were added. The same set of exon sequences was analysed for a recombination detection using RDP4 software [43] and no evidence of recombination was found.

Genetic differentiation among populations was quantified by computing pairwise *F*_ST_ (conventional F-statistics from haplotype frequencies). Slatkin’s linearized *F*_ST_ estimates were tested for correlation with pairwise measures of the natural logarithm of the geographic distance using Mantel’s test [44]. In order to evaluate if some populations contribute differently than others to the average *F*_ST_, population specific *F*_ST_ indices were also calculated [45].

In order to estimate the total percentage of variance attributable to differences between species and among geographic areas (western and eastern Africa), a standard analysis of molecular variance AMOVA was performed with 5000 permutations [46]. These estimates were obtained with Arlequin version 3.11 [47] using the complete sequence for 70 individuals.

Sequential Bonferroni corrections adjusted critical probability values for multiple tests to minimize type I errors [48].

## Results

We obtained 70 sequences of 1335 bp. Twenty-four of the mosquitoes corresponded to *An. coluzzii* from Angola, Ghana-Okyereko and Guinea-Bissau. The remaining 46 samples corresponded to *An. gambiae* from Gabon, Ghana-Accra, Ghana-Okyereko, Guinea-Bissau, Mozambique and Tanzania. All sequences are available in the GenBank database under accession numbers: KX620787–KX620856.

### Genetic diversity and neutrality tests

The alignment of the 70 sequences resulted in 135 segregating sites defining 63 distinct haplotypes (Additional file 2: Table S2).

**Fig. 1:**
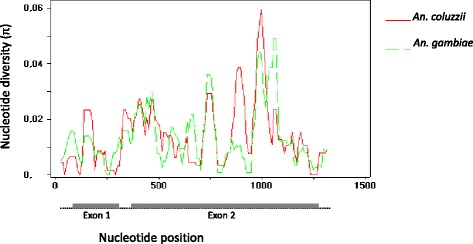
Nucleotide diversity (π) along the gene GNBP2. Exons are denoted by a *grey bold line*, introns are denoted by a *dashed line*. Sliding window was used (window length = 50 bp; sliding interval = 10 bp) adapted from DnaSP graphs

Summary diversity statistics are presented in Additional file 3: Table S3 The levels of haplotype diversity were very high and similar among populations and between species (0.911–1.000). The levels of nucleotide diversity compared between species are presented in Fig. 1.

**Fig. 2:**
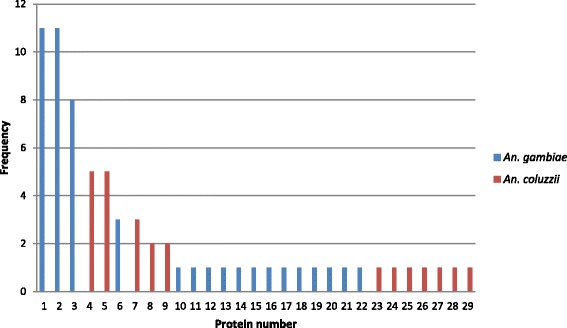
GNPB2 translated protein variation in *An. gambiae* (*s.s*.) and *An. coluzzii*

The translation of DNA sequences generated protein sequences with 391 amino acids. In GNBPB2, protein diversity was large. We obtained 17 proteins for *An. gambiae*, with 13 showing a frequency equal to one. The most common proteins had a frequency of 11, accounting for 24 % of the proteins detected. In *An. coluzzii,* we obtained 12 proteins, seven of which showed a frequency equal to one. The most common proteins had a frequency of 8, accounting for 24 % of the proteins detected (Fig. 2).

For the total sequence, all neutrality tests showed negative results (non-significant, Additional file 3: Table S3). However, significant negative values for Fu and Li’s D* and F* were detected when exon 1 was analysed separately. Furthermore, the D* value for the exon 2 was also significantly negative, over all populations. Considering each population individually, a significant positive value of Tajima’s D test was obtained for the intron section in mosquitoes from Angola.

**Fig. 3:**
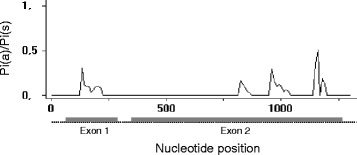
π(a)/π(s) ratios along the gene GNBP2. Exons are denoted by a *grey bold line*, introns are a *dashed line*. Sliding window was used (window length = 50 bp; sliding interval = 10 bp) adapted from DnaSP graphs

If most non-synonymous mutations are deleterious, then the rate of non-synonymous evolution will be lower than the neutral rate, resulting in π(a)/π(s) and dN/dS ratios < 1 [49]. Since our data revealed π(a)/π(s) ratio values lower than one, this suggests negative or purifying selection (Fig. 3). This ratio was particularly low in exon 2 (Additional file 3: Table S3).

From the dN/dS test performed with HyPhy, in the whole coding region we found two positively selected sites and 45 negatively selected sites, mainly evident in exon 2 (with only one site under positive selection and 40 sites under negative selection). As for exon 1, the selection signature was negligible (one site positively selected and another negatively selected).

When we focused on exon 2 by analysing more sequences from GenBank in a new alignment of 101 sequences, the number of negatively selected sites increased to 60, and with only two sites under positive selection.

### Genetic differentiation and population structure

Global *F*_ST_ among geographic locations was 0.018 (*P* < 0.003, Table 2) when the complete sequence was analysed, and 0.021 (*P* < 0.003) when only the coding regions (exon 1 and 2) were considered. The results presented hereafter refer to the whole sequence.

**Table 2:** Population specific *F*_ST_ indices for the complete sequence of *GNBPB2* gene analysed in *Anopheles gambiae* (*s.s*.) and *A. coluzii* from six African countries

Population	Species	*n*	*F*_ST_
Gabon	*An. gambiae*	9	0.013
Ghana (Accra)	*An. gambiae*	7	0.018
Ghana (Okyereko)	*An. gambiae*	6	0.021
Ghana (Okyereko)	*An. coluzzii*	6	0.021
Guinea Bissau	*An. coluzzii*	12	0.009
Guinea Bissau	*An. gambiae*	7	0.018
Angola	*An. coluzzii*	6	0.030
Mozambique	*An. gambiae*	10	0.020
Tanzania	*An. gambiae*	7	0.024
Global *F*_ST_ among populations		70	0.018

*Abbreviations*: *n* number of mosquitoes analysed, *F*_ST_ fixation index, a measure of population differentiation due to genetic structure

The pairwise differentiation (*F*_ST_) estimates ranged from 0 to 0.066, and all comparisons were non-significant (after Bonferroni correction). The same pattern was obtained when the species status was also taken into account, e.g. samples from Guinea Bissau and Ghana (Okyereko) were divided in two, one corresponding to *An. coluzzii* individuals and the other to *An. gambiae* (*s.s*.). The overall *F*_ST_ between species was 0.006 (*P* = 0.027).

We detected a significant correlation between genetic distance (Slatkin’s linearized FST) and geographic distance (Mantel test: *r* = 0.46, *P* = 0.02). Population specific FST indices for *An. coluzzii* ranged between 0.009 (Guinea Bissau) and 0.030 (Angola), while for *An. gambiae* specific FST values ranged between 0.013 (Gabon) and 0.024 (Tanzania) (Table 2).

The partition of molecular variance was 0.14 % of total variance (*P* ≥ 0.05) between species and 1.75 % (*P* < 0.01 in Table 3) among sample sites. On the other hand, when the groups were defined based on geographic areas: (E) East Africa (Mozambique and Tanzania) and (W) West Africa (all the rest), independent of species status, the molecular variance among groups increased to 2.26 % (*P* = 0.03), and the variance among sample sites was reduced to 0.85 % (*P ≥* 0.05). Furthermore, the combination with the maximum variation among groups (2.75 %, *P* < 0.01) was the one comprising three groups: Angola, East Africa and West Africa without Angola (Table 3).

**Table 3:** Partition of genetic variation based on conventional *F-*statistic from haplotype frequencies (AMOVA) for *Anopheles gambiae* (*s.s*.) and *A. coluzzii* over six African countries

Tested groups	Source of variation	Degrees of freedom	% of variation	*P*-value
Two species [*An. coluzzii & An. gambiae* (*s.s*.)]	Among groups	1	0.14	ns*
	Among populations within groups	7	1.75	0.006
	Within populations	61	98.11	0.001
Two geographical regions (W & E)	Among groups	1	2.26	0.030
	Among populations within groups	7	0.86	ns
	Within populations	61	96.89	0.002
Three geographical regions (W & E & Angola)	Among groups	2	2.75	0.004
	Among populations within groups	6	0.29	ns
	Within populations	61	96.96	0.002

*P-values ≥0.05 were considered non-significant (ns); *W* West Africa, *E* East Africa

## Discussion

The levels of nucleotide diversity for *GNBPB2* detected in this study are comparable with the GNBP gene reported by Lehmann et al. [30] and other immune-related genes in *An. gambiae* [24, 27, 32] and *Drosophila melanogaster* [50, 51]. As an exception, Morlais et al. [24] found levels of nucleotide diversity for *GNBPB1* ten times smaller (*Π* = 0.0016) than the values of *GNBPB2*, but this work was based on the analysis of laboratory strains rather than wild populations so that comparisons may not be straightforward. The patterns of high protein variation in *GNBPB2*, and high haplotype diversity in exon 2 may be consistent with diversifying selection, a mode of selection that maintains high levels of diversity (e.g. MHC genes in mammals [52]) and fits well with the role of immune recognition [53]. This selection for hyper-variability was not excluded for the *GNBP* gene in the study from Lehman et al. [30] that also presented large protein diversity.

Our study showed very low levels of genetic differentiation between species (*F*_ST_ = 0.006, *P* = 0.027), when compared with values obtained in other population studies in several immune-related genes [21, 22]. Unlike these studies where only samples from one village were used (from Cameroon in [21] and Burkina Faso in [22]), we sampled mosquitoes from nine countries, ranging from Guinea Bissau in western Africa to Mozambique in the Southeast of the continent. Overall, the effect of geographical distance among populations was more decisive in our study than that of species status. Indeed, the genetic discontinuity between West and East Africa accounts for 2.2 % (*P* = 0.03) of the total variance (Table 3), while the hierarchical genetic diversity analysis revealed that 0.14 % (*P* > 0.05) of the total variance arose from differences between the two species. Furthermore, we also detected a pattern of isolation by distance made evident by the significant positive correlation between genetic differentiation and geographical distance. This scenario has already been reported for *An. gambiae* in an extensive analysis of neutral markers on a large geographic scale by [54].

In terms of genetic structure, the sample of *An. coluzzii* from Angola stands out in this study. The variance among groups was maximized when three groups of samples were defined: West Africa, East Africa and Angola (2.76 % of variation, *P* = 0.01, Table 3). The population from Angola is the most differentiated population with the highest specific *F*_ST_ index (0.030, Table 2). This value is 1.7 times the global *F*_ST_ among populations, and is higher than the indices calculated for the most eastern populations (Mozambique and Tanzania). Such differential contribution to the average *F*_ST_ may suggest special evolutionary constraints in the population [55]. In fact, in Angola a significant positive value of the D Tajima’s neutrality test [39] in the intron section of the *GNBPB2* (Additional file 3: Table S3) was detected, which signifies an excess of intermediate frequency polymorphisms, indicating a possible decrease in population size.

Overall our findings may reflect a population structuring associated with different African biomes as reported by Pinto et al. [56]. These authors analysed *An. gambiae* samples from 12 African countries with 13 microsatellite loci and reported a strong population structuring within *An. coluzzii*, which was divided into three distinct genetic clusters (west, central, and southern Africa). These clusters were associated with the central African rainforest belt and northern and southern savannah biomes, suggesting limited gene flow between them. Furthermore, a study based on sequence analysis of an X-linked locus revealed that the majority of *An. coluzzii* individuals in Angola had a 16-bp insertion that was fixed in *An. gambiae* but absent in *An. coluzzii* individuals from west and central Africa [57], a finding that suggests interspecific introgression may have occurred in this geographical region.

The results of neutrality tests were generally variable and non-significant. Because both, selective events or demographic changes can produce similar deviations from neutrality in these tests [58] we used also the dN/dS ratio test that is not sensitive to demographic events, to help in the detection of selection effects. In both exons, dN/dS was <1, which signifies a rate of non-synonymous evolution lower than neutral rate, due to most non-synonymous mutations being deleterious (i.e. purifying selection) [49]. Indeed, a strong signature of purifying selection was detected essentially in exon 2, and further confirmed by a joint analysis of other sequences of exon 2 of *GNBPB2* available from previous studies [21, 34]. This is concordant with the majority of *Anopheles* immune related genes, which are also under purifying selection (e.g. [29, 30, 32]). Overall, this suggests functional constraints possibly associated with the immunoregulatory role of this gene.

## Conclusions

The present paper expands our limited knowledge about the gene *GNBPB2* with a population genetics approach in the two main malaria vectors, *An. gambiae* (*s.s*.) and *An. coluzzii*, over a wide geographic area in Africa. Our study showed that *GNBPB2* is similar in the two species. On the other hand, the variability of the gene is differentiated according to the geographic distance of different populations in the African continent. Generally, the selection tests results are consistent with most of the studies that have addressed questions regarding the evolution and genetic diversity of *Anopheles sp.* innate immunity genes involved in *Plasmodium* infection. Purifying selection was found to be the most common form of selection operating on these genes [21, 25–34], but diversifying selection should not be excluded. Specifically, Lehmann et al. [30] confirmed similar selective effects on the GNBP gene on a contemporary time scale.

## Additional files

Additional file 1: Table S1. Mosquito samples used in this study [59, 60]. (DOCX 18 kb) Additional file 2: Table 2. Alignment of polymorphic positions in GNBP2 after exclusion of all gaps (indels). Dots indicate identity with corresponding base of the first sequence. Position number is indicated above each base and species affiliation on the left of each sequence. (XLSX 56 kb) Additional file 3: Table S3. Genetic diversity and neutrality tests for the GNBPB2 gene in *An. gambiae* (s.s.) and *An. coluzzii*. (XLSX 32 kb)

## References

[1] Osta MA, Christophides GK, Vlachou D, Kafatos FC (2004). Innate immunity in the malaria vector *Anopheles gambiae*: comparative and functional genomics. J Exp Biol.

[2] Meister S, Kanzok SM, Zheng XL, Luna C, Li TR, Hoa NT (2005). Immune signaling pathways regulating bacterial and malaria parasite infection of the mosquito *Anopheles gambiae*. Proc Natl Acad Sci U S A.

[3] Holt R, Subramanian G, Halpern A, Sutton G, Charlab R, Nusskern D (2002). The genome sequence of the malaria mosquito *Anopheles gambiae*. Science.

[4] Mongin E, Louis C, Holt RA, Birney E, Collins FH (2004). The *Anopheles gambiae* genome: an update. Trends Parasitol.

[5] Cirimotich CM, Dong Y, Garver LS, Sim S, Dimopoulos G (2010). Mosquito immune defenses against *Plasmodium* infection. Dev Comp Immunol.

[6] Yassine H, Osta MA (2010). *Anopheles gambiae* innate immunity. Cell Microbiol.

[7] Christophides G, Zdobnov E, Barillas-Mury C, Birney E, Blandin S, Blass C (2002). Immunity-related genes and gene families in *Anopheles gambiae*. Science.

[8] Dimopoulos G, Richman A, Müller H-M, Kafatos FC (1997). Molecular immune responses of the mosquito *Anopheles gambiae* to bacteria and malaria parasites. Proc Natl Acad Sci U S A.

[9] Richman AM (1997). *Plasmodium* activates the innate immune response of *Anopheles gambiae* mosquitoes. Embo J.

[10] Tahar R, Boudin C, Thiery I, Bourgouin C (2002). Immune response of *Anopheles gambiae* to the early sporogonic stages of the human malaria parasite *Plasmodium* falciparum. Embo J.

[11] Aguilar R, Jedlicka AE, Mintz M, Mahairaki V, Scott AL, Dimopoulos G (2005). Global gene expression analysis of *Anopheles gambiae* responses to microbial challenge. Insect Biochem Molec Biol.

[12] Dong Y, Aguilar R, Xi Z, Warr E, Mongin E, Dimopoulos G (2006). *Anopheles gambiae* immune responses to human and rodent *Plasmodium* parasite species. PLoS Pathog.

[13] Warr E, Das S, Dong Y, Dimopoulos G (2008). The Gram-negative bacteria-binding protein gene family: its role in the innate immune system of *Anopheles gambiae* and in anti-*Plasmodium* defence. Insect Mol Biol.

[14] Hillyer JF. Mosquito immunity. In: Söderhäll K, editor. Invertebrate Immunity. Chapter 12: Landes Bioscience and Springer Science+Business Media; 2010. p. 218–238

[15] Dimopoulos G, Seeley D, Wolf A, Kafatos FC (1998). Malaria infection of the mosquito *Anopheles gambiae* activates immune-responsive genes during critical transition stages of the parasite life cycle. Embo J.

[16] Hughes GL, Ren X, Ramirez JL, Sakamoto JM, Bailey JA, Jedlicka AE, Rasgon JL. *Wolbachia* infections in *Anopheles gambiae* cells: transcriptomic characterization of a novel host-symbiont interaction. PLoS Pathog. 2011;7(2):e1001296. 10.1371/journal.ppat.1001296 PMC3040664 21379333

[17] Coetzee M, Hunt RH, Wilkerson R, della Torre A, Coulibaly MB, Besansky NJ. *Anopheles coluzzi*i and *Anopheles amharicus*, new members of the *Anopheles gambiae* complex. Zootaxa. 2013;3619:246-74. 26131476

[18] Turner T, Hahn M, Nuzhdin S (2005). Genomic islands of speciation in *Anopheles gambiae*. PLoS Biol.

[19] White BJ, Cheng C, Simard F, Costantini C, Besansky NJ (2010). Genetic association of physically unlinked islands of genomic divergence in incipient species of *Anopheles gambiae*. Mol Ecol.

[20] Weetman D, Wilding CS, Steen K, Pinto J, Donnelly MJ (2012). Gene flow-dependent genomic divergence between *Anopheles gambiae* M and S forms. Mol Biol Evol.

[21] Cohuet A, Krishnakumar S, Simard F, Morlais I, Koutsos A, Fontenille D (2008). SNP discovery and molecular evolution in *Anopheles gambiae*, with special emphasis on innate immune system. BMC Genomics.

[22] Crawford JE, Bischoff E, Garnier T, Gneme A, Eiglmeier K, Holm I (2012). Evidence for population-specific positive selection on immune genes of *Anopheles gambiae*. G3.

[23] White BJ, Lawniczak MKN, Cheng C, Coulibaly MB, Wilson MD, Sagnon NF (2011). Adaptive divergence between incipient species of *Anopheles gambiae* increases resistance to *Plasmodium*. Proc Natl Acad Sci U S A.

[24] Morlais I, Poncon N, Simard F, Cohuet A, Fontenille D (2004). Intraspecific nucleotide variation in *Anopheles gambiae*: new insights into the biology of malaria vectors. Am J Trop Med Hyg.

[25] Obbard DJ, Linton Y-M, Jiggins FM, Yan G, Little TJ (2007). Population genetics of *Plasmodium* resistance genes in *Anopheles gambiae*: no evidence for strong selection. Mol Ecol.

[26] Slotman MA, Parmakelis A, Marshall JC, Awono-Ambene PH, Antonio-Nkondjo C, Simard F (2007). Patterns of selection in anti-malarial immune genes in malaria vectors: evidence for adaptive evolution in LRIM1 in *Anopheles* arabiensis. PLoS One.

[27] Simard F, Licht M, Besansky N, Lehmann T (2007). Polymorphism at the defensin gene in the *Anopheles gambiae* complex: testing different selection hypotheses. Infect Genet Evol.

[28] Obbard DJ, Callister DM, Jiggins FM, Soares DC, Yan G, J T, Little TJ. The evolution of TEP1, an exceptionally polymorphic immunity gene in *Anopheles gambiae*. BMC Evol Biol. 2008;8(274) 10.1186/1471-2148-8-274 PMC2576239 18840262

[29] Parmakelis A, Slotman M, Marshall J, Awono-Ambene P, Antonio-Nkondjio C, Simard F, Caccone A, Powell J (2008). The molecular evolution of four anti-malarial immune genes in the *Anopheles gambiae* species complex. BMC Evol Biol.

[30] Lehmann T, Hume JC, Licht M, Burns CS, Wollenberg K, Simard F, Ribeiro JM (2009). Molecular evolution of immune genes in the malaria mosquito *Anopheles gambiae*. PLoS One.

[31] Obbard D, Welch J, Little T (2009). Inferring selection in the *Anopheles gambiae* species complex: an example from immune-related serine protease inhibitors. Malar J.

[32] Mendes C, Felix R, Sousa A-M, Lamego J, Charlwood D, do Rosario V (2010). Molecular evolution of the three short PGRPs of the malaria vectors *Anopheles gambiae* and *Anopheles arabiensis* in East Africa. BMC Evol Biol.

[33] Parmakelis A, Moustaka M, Poulakakis N, Louis C, Slotman MA, Marshall JC (2010). *Anopheles* immune genes and amino acid sites evolving under the effect of positive selection. PLoS One.

[34] Mendes AM, Schlegelmilch T, Cohuet A, Awono-Ambene P, De Iorio M, Fontenille D (2008). Conserved mosquito/parasite interactions affect development of *Plasmodium**falciparum* in Africa. PLoS Pathog.

[35] Collins F, Finnerty V, Petrarca V (1988). Ribosomal DNA-probes differentiate five cryptic species in the *Anopheles gambiae* complex. Parassitologia.

[36] Favia G, della Torre A, Bagayoko M, Lanfrancotti A, Sagnon N, Toure YT, Coluzzi M (1997). Molecular identification of sympatric chromosomal forms of *Anopheles gambiae* and further evidence of their reproductive isolation. Insect Mol Biol.

[37] Hall T (1999). BioEdit: a user friendly biological sequence alignment editor and analysis program for Windows 95/98/NT. Nucleic Acids Symp Ser.

[38] Librado P, Rozas J (2009). DnaSP v5: a software for comprehensive analysis of DNA polymorphism data. Bioinformatics.

[39] Tajima F (1989). Statistical method for testing the neutral mutation hypothesis by DNA polymorphism. Genetics.

[40] Fu YX, Li WH (1993). Statistical tests of neutrality of mutations. Genetics.

[41] Fu Y (1997). Statistical tests of neutrality of mutations against population growth, hitchhiking and background selection. Genetics.

[42] Pond SLK, Muse SV, Gail M, Krickeberg K, Samet J, Tsiatis A, Wong W (2005). HyPhy: hypothesis testing using phylogenies. Statistical methods in molecular evolution.

[43] Martin DP, Murrell B, Golden M, Khoosal A, Muhire B (2015). RDP4: detection and analysis of recombination patterns in virus genomes. Virus Evol.

[44] Smouse PE, Long JC, Sokal RR (1986). Multiple regression and correlation extensions of the Mantel Test of matrix correspondence. Syst Zool.

[45] Weir B, Cockerham C (1984). Estimating F-statistics for the analysis of population structure. Evolution.

[46] Excoffier L, Smouse P, Quattro J (1992). Analysis of molecular variance inferred from metric distances among DNA haplotypes: application to human mitochondrial DNA restriction data. Genetics.

[47] Excoffier L, Laval G, Schneider S (2005). Arlequin ver. 3.0: An integrated software package for population genetics data analysis. Evol Bioinform Online.

[48] Rice WR (1989). Analysis tables of statistical tests. Evolution.

[49] Ford M (2002). Applications of selective neutrality tests to molecular ecology. Mol Ecol.

[50] Labate JA, Biermann CH, Eanes WF (1999). Nucleotide variation at the runt locus in *Drosophila melanogaster* and *Drosophila simulans*. Mol Biol Evol.

[51] Jiggins F, Hurst G (2003). The evolution of parasite recognition genes in the innate immune system: purifying selection on *Drosophila melanogaster* peptidoglycan recognition proteins. J Mol Evol.

[52] Hughes AL, Nei M (1988). Pattern of nucleotide substitution at major histocompatibility complex class I loci reveals overdominant selection. Nature.

[53] Gilbert SC, Plebanski M, Gupta S, Morris J, Cox M, Aidoo M (1998). Association of malaria parasite population structure, HLA, and immunological antagonism. Science.

[54] Lehmann T, Licht M, Elissa N, Maega B, Chimumbwa J, Watsenga F (2003). Population structure of *Anopheles gambiae* in Africa. J Heredity.

[55] Weir BS, Hill WG (2002). Estimating F-statistics. Annu Rev Genet.

[56] Pinto J, Egyir-Yawson A, Vicente J, Gomes B, Santolamazza F, Moreno M (2013). Geographic population structure of the African malaria vector *Anopheles gambiae* suggests a role for the forest-savannah biome transition as a barrier to gene flow. Evol Appl.

[57] Choi KS, Townson H (2012). Evidence for X-linked introgression between molecular forms of *Anopheles gambiae* from Angola. Med Vet Entomol.

[58] Ramirez-Soriano A, Ramos-Onsins SE, Rozas J, Calafell F, Navarro A (2008). Statistical power analysis of neutrality tests under demographic expansions, contractions and bottlenecks with recombination. Genetics.

[59] Arez AP, Pinto J, Pålsson K, Snounou G, Jaenson TGT, Rosário V (2003). Transmission of mixed *Plasmodium* species and *Plasmodium**falciparum* genotypes. Am J Trop Med Hyg.

[60] Drakeley C, Schellenberg D, Kihonda J, Sousa CA, Arez AP, Lopes D (2003). An estimation of the entomological inoculation rate for Ifakara: a semi-urban area in a region of intense malaria transmission in Tanzania. Trop Med Int Health.

