# Supplementary material for: Molecular evolution and population genetics of a Gram-negative binding protein gene in the malaria vector Anopheles gambiae (sensu lato)
Source: Parasit Vectors. 2016 Sep 23;9:515. doi: 10.1186/s13071-016-1800-2 (PMC5034674; doi:10.1186/s13071-016-1800-2)
Supplement: Additional file 1: Table S1. — Mosquito samples used in this study [59, 60]. (DOCX 18 kb) [file 13071_2016_1800_MOESM1_ESM.docx]

# Additional file 1

# Table S1 Mosquito samples used in this study

| **Country** | **Locality** | **Geographic coordinates** | **Year** | **Collection method** | ***n*** | **Reference** |
| --- | --- | --- | --- | --- | --- | --- |
| Guinea-Bissau | Bissau | 11.8911, -15.5820 | 1996 | IR | 19 | [[59](#_ENREF_59)] |
| Ghana | Accra | 5.5441, -0.2386 | 2006 | IR | 7 | [[43](#_ENREF_43)] |
|  | Okyereko | 5.4145, -0.6045 | 2006 | IR | 12 | [[43](#_ENREF_43)] |
| Gabon | Dienga | -1.8470, 12.6920 | 1999 | LC | 9 | [[43](#_ENREF_43)] |
| Angola | Luanda | -8.8214, 13.2911 | 2002 | IR | 6 | [[43](#_ENREF_43)] |
| Tanzania | Ifakara | -8.1399, 36.6817 | 2003 | LT | 7 | [[60](#_ENREF_60)] |
| Mozambique | Furvela | -23.7162;,35.2986 | 2004 | LT | 10 | [[43](#_ENREF_43)] |

*Abbreviations*: LC, landing captures; IR, indoor resting capture; CDC light traps; n, number of mosquitoes analysed in the present study
