# Supplementary material for: Molecular evolution and population genetics of a Gram-negative binding protein gene in the malaria vector Anopheles gambiae (sensu lato)
Source: Parasit Vectors. 2016 Sep 23;9:515. doi: 10.1186/s13071-016-1800-2 (PMC5034674; doi:10.1186/s13071-016-1800-2)
Supplement: Additional file 3: Table S3. — Genetic diversity and neutrality tests for the GNBPB2 gene in An. gambiae (s.s.) and An. coluzzii. (XLSX 32 kb) [file 13071_2016_1800_MOESM3_ESM.docx]

# Additional file 3

# Table S3 Genetic diversity and neutrality tests for the GNBPB2 gene in *An. gambiae* (*s.s.*) and *An. coluzzii*

|  | | | | | | | | | | | |
| --- | --- | --- | --- | --- | --- | --- | --- | --- | --- | --- | --- |
| **DNA** | **Population** | **Angola** | **Gabon** | **Ghana-A** | **Ghana-O** | **Guinea Bissau** | **Mozambique** | **Tanzania** | ***An. coluzzi*** | ***An. gambiae* (*s.s*.)** | **All individuals** |
| **region** | **N** | 6 | 9 | 7 | 12 | 19 | 10 | 7 | 24 | 46 | 70 |
|  |  |  |  |  |  |  |  |  |  |  |  |
|  | **S** | 1 | 3 | 6 | 2 | 0 | 1 | 5 | 3 | 11 | 10 |
|  | **Hap** | 2 | 3 | 3 | 3 | 1 | 2 | 3 | 4 | 5 | 7 |
|  | **Hd** | 0.600 | 0.556 | 0.667 | 0.318 | 0.000 | 0.556 | 0.667 | 0.370 | 0.244 | 0.287 |
| **5' upstream** | ***Π*** | 0.007 | 0.010 | 0.033 | 0.006 | 0.000 | 0.007 | 0.021 | 0.006 | 0.009 | 0.008 |
| **(1-82 bp)** | **TajimaD** | 1.445 | -0.936 | 0.452 | -0.850 | n.d. | 1.464 | -0.793 | -1.041 | -2.048* | -1.868* |
|  | **FuLiD*** | 1.053 | -0.726 | 0.951 | -0.374 | n.d. | 0.804 | -0.969 | -0.189 | -0.812 | 0.070 |
|  | **FuLiF*** | 1.158 | -0.860 | 0.914 | -0.556 | n.d. | 1.069 | -1.010 | -0.494 | -1.434 | -0.696 |
|  | **FuFs** | 0.795 | 0.016 | 1.934 | -0.725 | n.a. | 1.096 | 1.014 | -1.534 | -0.902 | -3.215 |
|  |  |  |  |  |  |  |  |  |  |  |  |
| **Exon 1** | **S** | 3 | 7 | 5 | 8 | 8 | 4 | 5 | 8 | 14 | 17 |
| **(83-301)** | **Hap** | 2 | 7 | 5 | 8 | 8 | 3 | 4 | 9 | 15 | 19 |
|  | **Hd** | 0.600 | 0.917 | 0.905 | 0.894 | 0.860 | 0.689 | 0.714 | 0.880 | 0.824 | 0.814 |
|  | ***Π*** | 0.008 | 0.009 | 0.009 | 0.011 | 0.008 | 0.008 | 0.007 | 0.010 | 0.008 | 0.008 |
|  | **TajimaD** | 1.910 | -0.901 | -0.330 | -0.453 | -0.764 | 0.626 | -1.024 | 0.080 | -1.424 | -1.480 |
|  | **FuLiD*** | 1.396 | -0.535 | -0.370 | -0.531 | -0.325 | 1.239 | -0.969 | -0.456 | -1.298 | -2.486* |
|  | **FuLiF*** | 1.582 | -0.694 | -0.393 | -0.580 | -0.519 | 1.220 | -1.066 | -0.347 | -1.582 | -2.530* |
|  | **FuFs** | 2.759 | -3.706 | -1.654 | -3.410 | -2.766 | 1.516 | -0.538 | -2.378 | -8.874 | -12.347 |
|  | ***Π*(a) / *Π*(s)** |  |  |  |  |  |  |  | 0.226 | 0.105 | 0.108 |
|  | **dN/dS** |  |  |  |  |  |  |  |  |  | 0.500 |
|  | **(95% CI)** |  |  |  |  |  |  |  |  |  | (0.298 - 0.777) |
|  |  |  |  |  |  |  |  |  |  |  |  |
| **Intron** | **S** | 4 | 1 | 3 | 7 | 2 | 4 | 4 | 5 | 5 | 7 |
| **(302-371 bp)** | **Hap** | 2 | 2 | 3 | 6 | 3 | 3 | 3 | 4 | 6 | 7 |
|  | **Hd** | 0.600 | 0.500 | 0.667 | 0.758 | 0.205 | 0.689 | 0.667 | 0.656 | 0.644 | 0.653 |
|  | ***Π*** | 0.034 | 0.007 | 0.020 | 0.026 | 0.004 | 0.023 | 0.022 | 0.023 | 0.015 | 0.016 |
|  | **TajimaD** | 2.006* | 0.986 | 0.755 | -0.869 | -1.120 | 0.626 | -0.319 | 0.568 | -0.240 | -0.576 |
|  | **FuLiD*** | 1.467 | 0.840 | 0.389 | -1.320 | -0.574 | 1.239 | -0.069 | 1.166 | -0.795 | 0.392 |
|  | **FuLiF*** | 1.676 | 0.962 | 0.508 | -1.366 | -0.825 | 1.220 | -0.135 | 1.152 | -0.729 | 0.087 |
|  | **FuFs** | 3.430 | 0.849 | 0.668 | -1.607 | -1.152 | 1.516 | 0.789 | 1.404 | -1.048 | -1.201 |
|  |  |  |  |  |  |  |  |  |  |  |  |
| **Exon 2** | **S** | 17 | 37 | 38 | 50 | 68 | 25 | 35 | 67 | 74 | 101 |
| **(372-1327bp)** | **Hap** | 5 | 9 | 7 | 12 | 19 | 7 | 6 | 23 | 39 | 62 |
|  | **Hd** | 0.933 | 1.000 | 1.000 | 1.000 | 1.000 | 0.911 | 0.952 | 0.996 | 0.990 | 0.995 |
|  | **VarHd** | 0.015 | 0.003 | 0.006 | 0.001 | 0.000 | 0.006 | 0.009 | 0.000 | 0.000 | 0.000 |
|  | ***Π*** | 0.010 | 0.014 | 0.016 | 0.017 | 0.015 | 0.011 | 0.016 | 0.016 | 0.015 | 0.016 |
|  | **TajimaD** | 1.406 | 0.020 | -0.177 | -0.358 | -1.277 | 1.069 | 0.305 | -0.869 | -0.651 | -1.188 |
|  | **FuLiD*** | 1.118 | -0.283 | -0.218 | -0.193 | -1.657 | 0.936 | 0.111 | -1.337 | -1.447 | -2.363* |
|  | **FuLiF*** | 1.283 | -0.236 | -0.231 | -0.270 | -1.798 | 1.094 | 0.171 | -1.396 | -1.380 | -2.271 |
|  | **FuFs** | 0.667 | -2.097 | -0.845 | -3.375 | -8.669 | 1.059 | 0.899 | -9.806 | -18.875 | -33.634 |
|  | ***Π*(a) / *Π*(s)** |  |  |  |  |  |  |  | 0.024 | 0.022 | 0.022 |
|  | **dN/dS** |  |  |  |  |  |  |  |  |  | 0.030 |
|  | **(95% CI)** |  |  |  |  |  |  |  |  |  | (0.020 - 0.044) |
|  |  |  |  |  |  |  |  |  |  |  |  |
| **total** | **S** | 25 | 48 | 52 | 67 | 78 | 34 | 49 | 83 | 104 | 135 |
| **(1-1335 bp)** | **Hap** | 5 | 9 | 7 | 12 | 19 | 7 | 6 | 23 | 40 | 63 |
|  | **Hd** | 0.933 | 1.000 | 1.000 | 1.000 | 1.000 | 0.911 | 0.952 | 0.996 | 0.991 | 0.996 |
|  | **VarHd** | 0.015 | 0.003 | 0.006 | 0.001 | 0.000 | 0.006 | 0.009 | 0.000 | 0.000 | 0.000 |
|  | ***Π*** | 0.010 | 0.013 | 0.016 | 0.015 | 0.012 | 0.011 | 0.015 | 0.014 | 0.014 | 0.014 |
|  | **TajimaD** | 1.717 | -0.175 | -0.055 | -0.476 | -1.262 | 1.064 | -0.021 | -0.718 | -0.961 | -1.320 |
|  | **FuLiD*** | 1.320 | -0.339 | -0.051 | -0.403 | -1.533 | 1.124 | -0.154 | -1.069 | -1.488 | -2.216 |
|  | **FuLiF*** | 1.534 | -0.336 | -0.057 | -0.482 | -1.692 | 1.252 | -0.138 | -1.125 | -1.545 | -2.225 |
|  | **FuFs** | 1.368 | -1.652 | -0.367 | -2.678 | -7.814 | 1.795 | 1.362 | -7.858 | -17.138 | -42.192 |
|  | ***Π*(a) / *Π*(s)** |  |  |  |  |  |  |  | 0.040 | 0.029 | 0.029 |
|  | **dN/dS** |  |  |  |  |  |  |  |  |  | 0.052 |
|  | **(95% CI)** |  |  |  |  |  |  |  |  |  | (0.039 - 0.069) |
| **Genetic Diversity:** S number of Segregating Sites, Hap number of haplotypes, Hd Haplotype diversity *Π* Nucleotide diversity, *Π*(s) Synonymous Nucleotide Diversity, *Π*(a) Nonsynonymous Nucleotide Diversity. **Neutrality tests:** Tajima’s D (Tajima 1989), Fu and Li’s D* (Fu and Li 1993), Fu and Li’s F* (Fu and Li 1993), Fu’s Fs (Fu 1997) and their statistical significance: * *P* < 0.05;dN/dS: dN rate of nonsynonymous substitutions per nonsynonymous site dS rate of synonymous substitutions per synonymous site, CI confidence interval | | | | | | | | | | | |
